# Atomic Layer Deposition of Nickel Using Ni(dmamb)_2_ and ZnO Adhesion Layer Without Plasma

**DOI:** 10.1007/s41871-024-00238-5

**Published:** 2024-09-20

**Authors:** Kaiya Baker, Hayden Brown, Fisseha Gebre, Jiajun Xu

**Affiliations:** Center for Advanced Manufacturing in Space Technology and Applied Research (CAM-STAR), University of the District of Columbia, Washington, DC USA

**Keywords:** ZnO adhesion layer, Ni deposition layer, Atomic layer deposition, Characterization using XRD and XPS

## Abstract

In this study, a novel deposition technique that utilizes diethylzinc (C_4_H_10_ZnO) with H_2_O to form a ZnO adhesion layer was proposed. This technique was followed by the deposition of vaporized nickel(II) 1-dimethylamino-2-methyl-2-butoxide (Ni(dmamb)_2_) and H_2_ gas to facilitate the deposit of uniform layers of nickel on the ZnO adhesion layer using atomic layer deposition. Deposition temperatures ranged from 220 to 300 °C. Thickness, composition, and crystallographic structure results were analyzed using spectroscopic ellipsometry, scanning electron microscopy (SEM), X-ray photoelectron spectroscopy (XPS), and X-ray diffraction (XRD), respectively. An average growth rate of approximately 0.0105 angstroms per cycle at 260 °C was observed via ellipsometry. Uniform deposition of ZnO with less than 1% of Ni was displayed by utilizing the elemental analysis function via SEM, thereby providing high-quality images. XPS revealed ionizations consistent with nickel and ZnO through the kinetic and binding energies of each detected electron. XRD provided supplemental information regarding the validity of ZnO by exhibiting crystalline attributes, revealing the presence of its hexagonal wurtzite structure.

## Highlights

1. Depositing nickel onto a silicon wafer and characterizing it using various techniques.
2. Ni deposition without plasma formation using hydrogen as a reactant.
3. ZnO formation as an adhesion layer and temperature is a crucial parameter.

## Introduction

Atomic layer deposition (ALD), a form of chemical vapor deposition (CVD), is an emerging technique that enables the deposition of thin films on substrates for various applications. ALD involves surface-level monolayer deposition, allowing for highly controllable conformal deposition [1]. The execution of ALD involves the reaction of gaseous precursors to initiate a self-limiting reaction, resulting in a thin film on the surface area of the substrate [2]. The increasing prominence of ALD in the nanoengineering field enhances various applications due to its precise technology [3]. Reactions are driven to completion with every cycle, minimizing the randomness of precursor flux and kinematic collisions of particles [3]. This reduction in randomized variables allows for the smoothest possible granular layer for uniform deposition. Owing to these qualities, ALD is optimal for the nanomanufacturing of thin films in microtechnology used for conductive processes, diffusion barriers, and electro-optical properties.

In previous electrolytic deposition studies, powdered nickel has been deposited by reacting solid nickel with carbon monoxide gas to form nickel carbonyl gas, which is further heated to yield “pure” nickel powder [4]. The technique involves the sublimation and deposition of nickel in a plasma environment. However, despite producing a pure substance, the powder often contains numerous impurities that may have accumulated during the sublimation phase of the mechanism. These byproducts can pose additional toxicity concerns, diminish the effectiveness of the intended application of the project, and yield inaccurate experimental data, thereby affecting reproducibility. This project also catalyzes the reaction with a gaseous nickel precursor using ALD to minimize impurities. The gas atomization process yields small particle sizes (upon appropriate thermal conditions) and narrow distributions, thus reducing the chances of impurities once pressurized in an atomic chamber. Powders will then rapidly solidify once they react with additional precursors.

Nickel deposition studies utilizing the atomic layer or CVD have also produced thin films at extremely high temperatures exceeding 300 °C [5–7]. Elevated temperatures in CVD may lead to substrate degradation, increased diffusion rates for the reacting precursors on the substrate, and heightened stress formation. The innovative approach of utilizing H_2_ gas as a precursor enables improved reduction chemistry by facilitating the donation of electrons, particularly because metal surfaces are typically unreactive [8]. This technique circumvents the intrinsic nature of nickel, which forms strong metallic bonds within its lattice structure, resulting in high stability and low reactivity [8].

This research aims to deposit nickel using a gaseous nickel precursor with minimal impurities while employing the lowest possible thermal activation energy to enhance product efficiency. As the seed layer, Ni facilitates the growth of carbon nanotubes on the surface of optics [9], enabling the incorporation of unique optical properties into spacecraft development.

## Materials and Methods

For this research, a commercial ALD 150LE™ chamber by Kurt J. Lesker, which includes a purely thermal process chamber configuration, was used. The chamber incorporates a perpendicular flow and showerhead design for uniform precursor dispersion and delivery. Precursors for nickel deposition were chosen due to previously reported procedures [9]. Diethylzinc is a highly pyrophoric liquid containing a boiling point of 118 °C at 760 Torr. Water was used as the co-reactant for diethylzinc, which has a boiling point of 100 °C at 760 Torr. Ni(dmamb)_2_ is a viscous liquid with a boiling point of 148 °C at 3.32 Torr. H_2_ gas, the co-reactant for Ni(dmamb)_2_, has a boiling point of − 259.16 °C at 760 Torr. All precursors contain properties sufficient for use in ALD. Si (100) and Si (111) crystallographic structure substrates were used for deposition and were cleaned thoroughly with acetone, isopropyl alcohol (IPA), and deionized water.

### Zinc Oxide Deposition

The reaction between DEZ and H_2_O proceeds in a multistep decomposition reaction as follows [8]:

1 $$\left( {{\text{C}}_{{\text{2}}} {\text{H}}_{{\text{5}}} } \right)_{{\text{2}}} {\text{Zn}} + {\text{H}}_{{\text{2}}} {\text{O}} \to {\text{C}}_{{\text{2}}} {\text{H}}_{{\text{5}}} {\text{ZnOH}} + {\text{C}}_{{\text{2}}} {\text{H}}_{{\text{6}}}$$

2 $${\text{C}}_{{\text{2}}} {\text{H}}_{{\text{5}}} {\text{ZnOH}} + \left( {{\text{C}}_{{\text{2}}} {\text{H}}_{{\text{5}}} } \right)_{{\text{2}}} {\text{Zn}} \to {\text{C}}_{{\text{2}}} {\text{H}}_{{\text{5}}} {\text{ZnOZnC}}_{{\text{2}}} {\text{H}}_{{\text{5}}} + {\text{C}}_{{\text{2}}} {\text{H}}_{{\text{6}}} .$$

3 $${\text{2C}}_{{\text{2}}} {\text{H}}_{{\text{5}}} {\text{ZnOH}} \to {\text{C}}_{{\text{2}}} {\text{H}}_{{\text{5}}} {\text{ZnOZnOH}} + {\text{C}}_{{\text{2}}} {\text{H}}_{{\text{6}}} .$$

Diethylzinc decomposes into monoethyl zinc when reacted with water [2, 10, 11]. The rate-limiting step includes the formation of ethane as a co-reactant and its further degradation into zinc oxide and zinc hydroxide molecules at sufficient temperatures [11–15]. Detailed formulas outlining the process parameters are shown below in Table 1.

**Table 1:** ZnO process parameters

Trial	Reactant A dose time (ms)	Purge time A (ms)	Reactant B dose time (ms)	Purge time B (ms)	Temp (°C)	# of cycles
1	6.5	10,000	50	10,000	150	200
2	6.5	10,000	50	10,000	150	200
3	13	5000	75	5000	150	200
4	13	5000	75	5000	150	200
5	15	7500	75	7500	150	200

### Nickel Deposition

The nickel deposition precursors, Ni(dmamb)_2_ and H_2_, yield solid nickel in the following reaction given proper thermal activation:

4 $${\text{C}}_{{{14}}} {\text{H}}_{{{32}}} {\text{N}}_{{2}} {\text{O}}_{{2}} {\text{Ni}} + {\text{H}}_{{2}} \to {\text{Ni(OH)}}_{{2}} + {\text{C}}_{{{14}}} {\text{H}}_{{{32}}} {\text{N}}_{{2}}$$

This reaction may produce several solid nickel variations, including NiO, Ni(OH)_2_, NiOOH, Ni-ZnO, NiC, Ni_2_Si, and pure Ni metal, as a result of interactions with Zn, H, O, Si, and C in a highly pressurized environment [11]. Within ALD, several side reactions exist between the precursor and the byproducts due to varying volatility and thermal stability in the atmosphere. The main reaction should yield notable thin films comprising predominantly Ni(OH)_2_. Initially, unreacted byproducts containing oxygen, hydrogen, silicon, carbon, and zinc will produce multiple Ni compounds originating from the source of the precursor, namely Ni(dmamb)_2_, which was contained in an ampoule at 110 °C to obtain adequate vapor pressure for the ALD reactor. One ALD cycle comprises four steps: precursor exposure, purging, reactant exposure, and purging once more with argon purging. The design of the experimental parameters is shown in Table 2.

**Table 2:** Ni process parameters

Trial	Reactant A dose time (ms)	Purge time A (ms)	Reactant B dose time (ms)	Purge time B (ms)	Temp (°C)	# of cycles
1	1000	10,000	6	120,000	220	200
2	1000	10,000	6	120,000	240	200
3	1000	10,000	6	120,000	260	200
4	1000	10,000	6	120,000	280	200
5	1000	10,000	6	120,000	300	200

Elemental analysis was performed with each trial using the energy-dispersive spectroscopy (EDS) function on the NanoScience Phenom Desktop scanning electron microscopy (SEM) machine. Images were captured using a mode of 10 kV and a secondary electron detector. The thickness of the nickel thin film was measured by the J.A. Woollam M-2000 DI spectroscopic ellipsometer. X-ray diffraction (XRD) was used for the microstructure analysis of deposited elements. The chemical composition of each element was investigated using X-ray photoelectron spectroscopy (XPS).

## Results and Discussions

### SEM Analysis

As shown in Table 2, a set of trials were performed to determine if temperatures within a range of 220–300 °C and an increased cycle count would facilitate Ni growth. Each trial comprised 200 cycles with a Ni dose time of 1000 ms, purge time of 10,000 ms, H_2_ dose time of 6 ms, and second purge time of 120,000 ms.

**Fig. 1:**
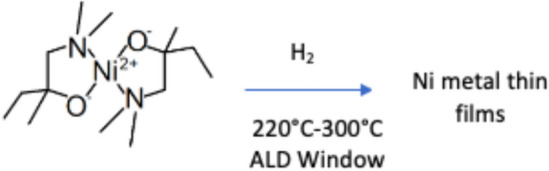
Diagram depicting the reaction mechanism of Ni(dmamb)_2_ and H_2_ yielding nickel thin films

Analysis of various deposition temperatures reveals substantial Ni deposition at 260 °C. Ni deposition requires a ZnO adhesion layer. Tables 3, 4, 5, 6 and 7 show the concentration of Zn and oxygen alongside Ni deposition. Thus, the high weight concentration of ZnO in Table 5 likely contributes to the increased Ni deposition because its stable hexagonal wurtzite structure and lattice parameters are optimal for Ni nanoparticle aggregation. At 220 °C and 240 °C, Zn and Ni have insufficient activation energy, incomplete precursor adsorption, or low surface mobility. At 280 °C and 300 °C, precursors could undergo thermal decomposition or desorption before their reaction. Elemental weight is optimal at 260 °C, where precursor growth is optimal for ZnO and Ni, thus reflecting the higher weight concentrations for each respective element in Table 5 (Fig. 1).

**Table 3:** EDS results at 220 °C

Element #	Element symbol	Element name	Weight concentration (%)
14	Si	Silicon	61.16
8	O	Oxygen	36.25
30	Zn	Zinc	1.70
28	Ni	Ni	0.88

**Table 4:** EDS results at 240 °C

Element #	Element symbol	Element name	Weight concentration (%)
14	Si	Silicon	50.77
8	O	Oxygen	31.30
30	Zn	Zinc	13.75
28	Ni	Ni	4.18

**Table 5:** EDS results at 260 °C

Element #	Element symbol	Element name	Weight concentration (%)
28	Ni	Ni	42.82
30	Zn	Zinc	38.35
8	O	Oxygen	16.44
14	Si	Silicon	2.39

**Table 6:** EDS results at 280 °C

Element #	Element symbol	Element name	Weight concentration (%)
14	Si	Silicon	80.70
30	Zn	Zinc	17.63
8	O	Oxygen	1.04
28	Ni	Ni	0.63

**Table 7:** EDS results at 300 °C

Element #	Element symbol	Element name	Weight concentration (%)
14	Si	Silicon	73.33
8	O	Oxygen	17.99
28	Ni	Ni	7.00
30	Zn	Zinc	1.68

**Fig. 2:**
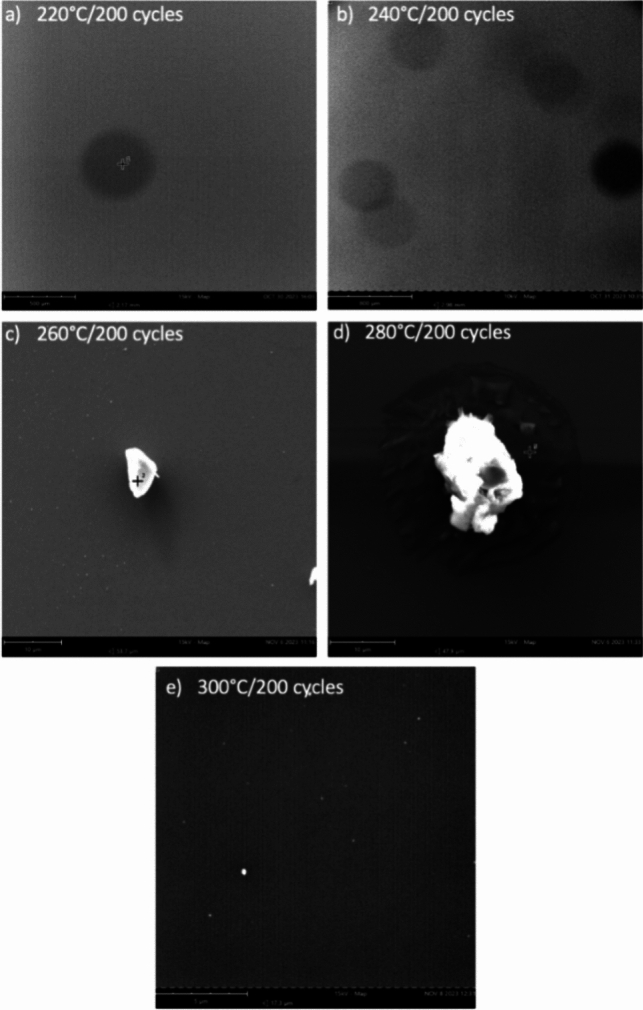
SEM images of Ni deposited on a silicon dioxide substrate at **a** 220 °C, **b** 240 °C, **c** 260 °C, **d** 280 °C, and **e** 300 °C for 200 cycles each using 15 kV mapping for image quality. The shapes depict the nucleation of Ni compounds at various sites atop the substrate

Figure 2 reveals slightly large globular growths of the elements mentioned in Tables 3–7 across the surface of the silicon wafer. Increased nucleation is observed at 220–300 °C. Figure 2a,b shows minute elemental growth due to relatively small weight concentrations of Zn and Ni in their elemental compositions. Heightened growth is found at 260 °C and 280 °C, shown in Fig. 2c,d, possibly revealing increased selective deposition at high temperatures. The parameters in Table 2 reveal increased Ni deposition; however, weight concentration in specific trials is displayed under 1%. Figure 2 also reflects the elemental changes from trials 1–5, with the larger nucleation at 260 °C (Fig. 2c) accounting for a weight concentration of Ni at 42.82%. Figure 2d illustrates an increase in the size of globular GROWTH. However, this change in size may not appear to be attributable to Ni composition, as the elemental analysis indicates significantly higher concentrations of Zn (17.63%) and Si (80.70%). Figure 2e follows the same trend as Fig. 2a,b. As previously mentioned, ZnO adhesion layer is necessary to catalyze Ni growth on the substrate. Therefore, the low concentrations in trials 1, 2, and 5 reveal a reduced Ni composition.

### XRD Analysis

XRD analysis was conducted to confirm the presence of Ni and ZnO via crystallographic structure, and raw files were analyzed using Profex Software 5.2.3.

**Fig. 3:**
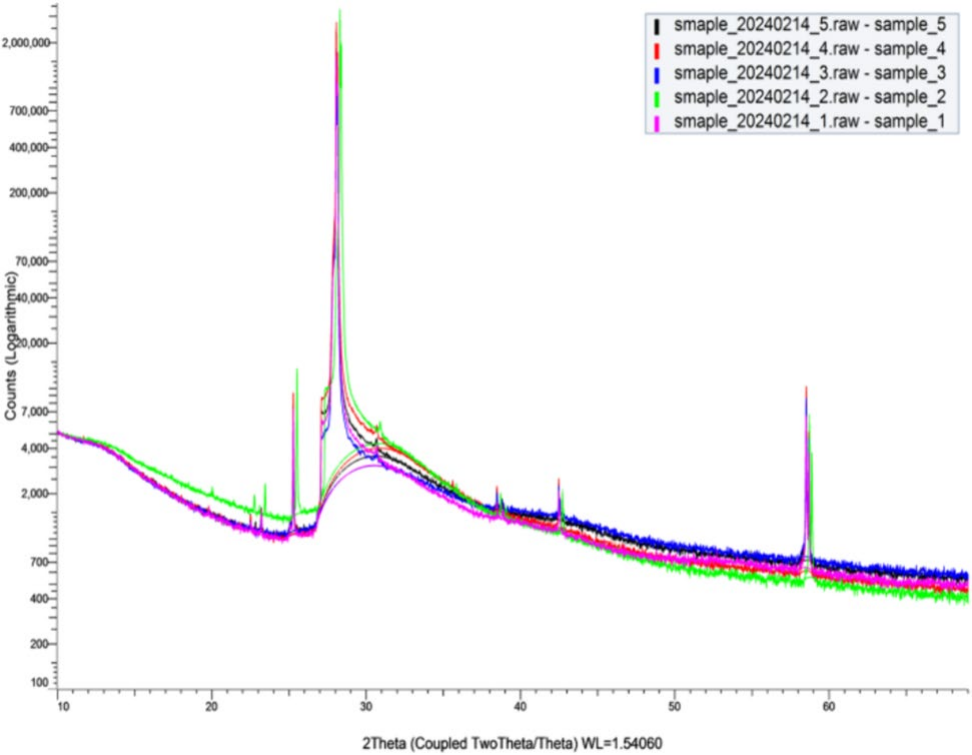
XRD results at 220 °C/200 cycles of Ni deposition

As shown in Fig. 3, the results at 220 °C display a notable intensity peak approximately between 15,000 and 40,000 counts between diffraction angles 25° and 30°. This finding indicates that a 111 lattice silicon substrate was used for deposition. A silicon 100 wafer maintains a flat, planar structure throughout its applications, enabling even distributions. A silicon 111 wafer holds a crystal orientation; due to its asymmetry, this wafer yields products with irregular edges. ZnO crystallizes on 100 silicon substrates as a hexagonal wurtzite (solid hexagonal) structure, making the 100 lattice silicon conformation ideal for deposition. The introduction of Ni nanoparticles results in a change of conformation to a hexagonal ring shape (with a hollow space inside). Ni was slightly detected using SEM/EDS. However, insignificant traces of Ni in the higher temperature trials remained undetected by X-rays due to its inability to properly diffract within inter-atomic spacing. ZnO nanoparticles were detected, as indicated by small peaks from 20° to 80° diffraction angles with intensities consistent with lattices, revealing a hexagonal wurtzite structure.

Tables 3–7 display the weight concentration of Ni for each trial. Trials 1, 2, 3, 4, and 5 reveal weight concentrations of 0.88%, 4.18%, 42.82%, 0.63%, and 7.00% at 220 °C, 240 °C, 260 °C, 280 °C, and 300 °C, respectively. Trials 1 and 4 both show the weight concentrations of Ni under 1%. However, this result may be due to the specific placement of the substrate in the gas chamber because some regions within the chamber contain higher amounts of deposition from the showerhead.

Tables 8, 9, 10, 11, and 12 show the XRD results of the Ni deposition at various temperatures for the same 200 cycles. Tables 9–12 show similar results to the first cycle with 220 °C in Table 8, likely indicating the substrate used was a silicon (111) wafer with minimal Ni deposition and a notable presence of ZnO. The results in Tables 9–12 reveal a similarity that assumes the Si(111) wafer is explained due to an intensity of 15,000–40,000 counts with a diffraction angle of approximately 28° (a characteristic of Si(111) substrate).

**Table 8:** XRD results at 220 °C/200 cycles of Ni deposition

Intensity	19,235
Distance (Å)	3.161
Diffraction angle 2*θ*	28.207°

**Table 9:** XRD results at 240 °C/200 cycles

Intensity	36,942
Distance (Å)	3.147
Diffraction angle 2*θ*	28.338°

**Table 10:** XRD results at 260 °C/200 cycles

Intensity	17,450
Distance (Å)	3.161
Diffraction angle 2*θ*	28.207°

**Table 11:** XRD results at 280 °C/200 cycles

Intensity	30,221
Distance (Å)	3.168
Diffraction angle 2*θ*	28.141°

**Table 12:** XRD results at 300 °C/200 cycles

Intensity	26,436
Distance (Å)	3.176
Diffraction angle 2*θ*	28.076°

### Ellipsometry Analysis

**Fig. 4:**
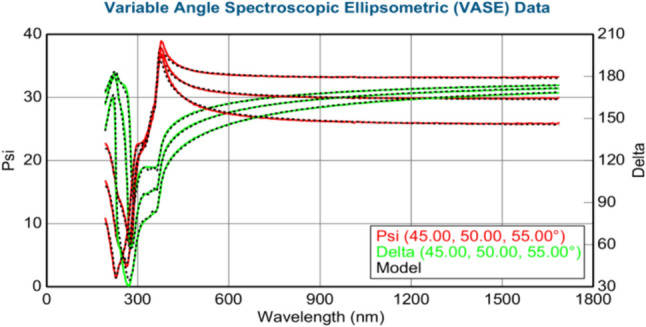
Graphical representation of ellipsometry results at 220 °C/200 cycles

**Fig. 5:**
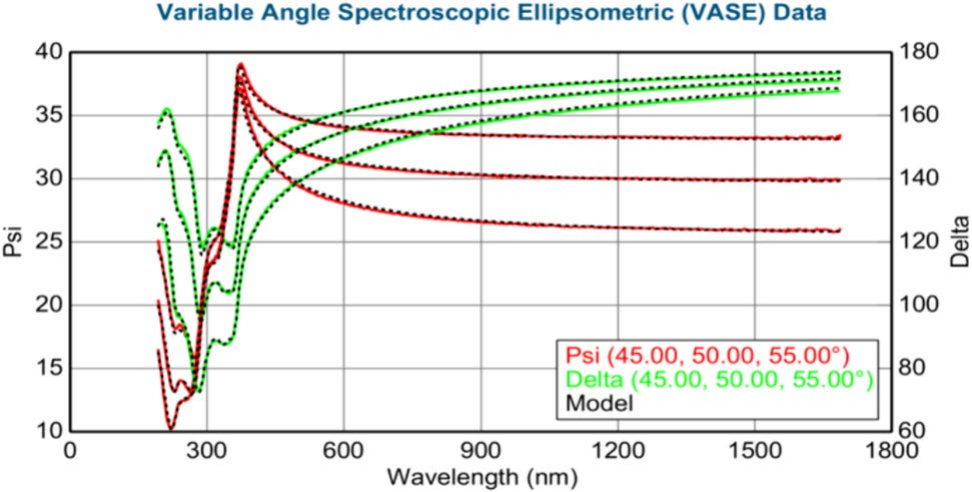
Ellipsometry results at 240 °C/200 cycles of Ni deposition

**Fig. 6:**
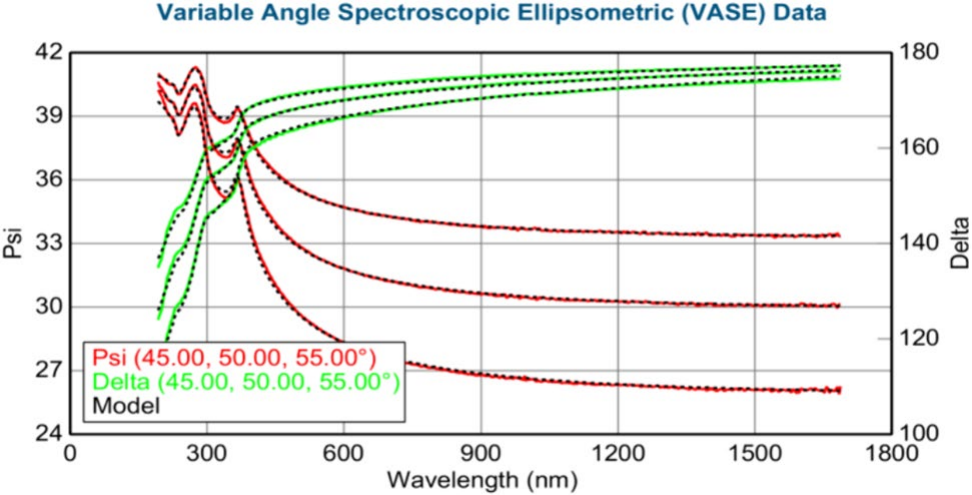
Ellipsometry results at 260 °C/200 cycles of Ni deposition

**Fig. 7:**
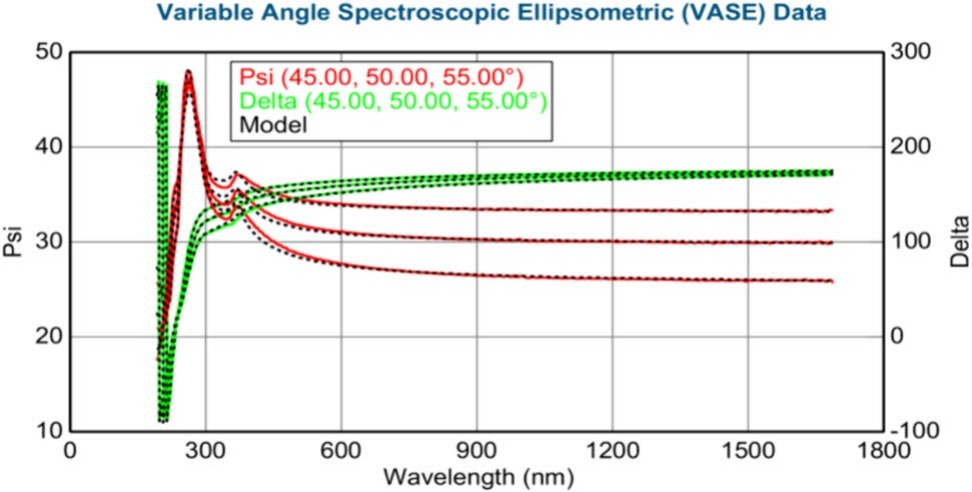
Ellipsometry results at 280 °C/200 cycles of Ni deposition

**Fig. 8:**
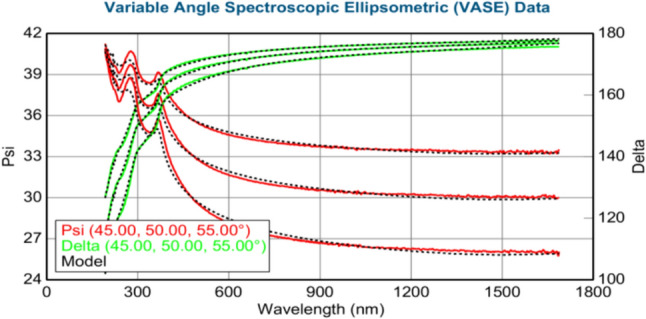
Ellipsometry results at 300 °C/200 cycles of Ni deposition

The thickness of the layers (in nm) was calculated using the J.A. Woollam M-2000 DI spectroscopic ellipsometer. Figures 3–8 show variable angle spectroscopic ellipsometry data, which characterize thin film surface material in wavelength vs. psi. Figure 3 reveals raw psi (represented in red) and delta values (represented in green), which describe the change in polarization that occurs when the measurement beam interacts with the surface of the substrate. The incident light beam contains electric fields parallel and perpendicular to the plane of incidence. Ellipsometry measures the two parameters; therefore, the thickness of each film and the index of refraction of each film can be determined from psi and delta, respectively. These variables are measured against multiple wavelengths to measure sample properties, thus best matching their properties to elements within their database.

Based on the psi and delta values of Fig. 4, the graph reveals the best match in the wavelength range for ZnO and Ni. In this measurement, the value for Ni is negative (− 0.04 nm) due to the algorithm of the ellipsometer, which is attempting to best fit the results with the database information of Ni. Moreover, obtaining negative values at low temperatures is possible because Ni growth is not uniform over the surface of the sample. At 220 °C, small amounts of Ni are detected; however, a substantial amount of ZnO (34.11 nm) is observed, which remains consistent with other forms of characterization previously reported in this study. The measured standard error (MSE) value is less than 10, calculated at 6.998, revealing highly accurate data collected by the ellipsometer, as shown in Table 13.

**Table 13:** XRD results at 220 °C/200 cycles of Ni deposition

MSE	6.998
Thickness 2: nickel (nm)	− 0.04 ± 0.041
Thickness 1: zinc oxide (nm)	34.11 ± 0.043
Average growth rate (Å/cycle)	− 0.002

Based on Fig. 5, at 240 °C, the thicknesses of ZnO and Ni have increased with ZnO, displaying 34.29 and 0.14 nm, respectively. The MSE value is 6.104, reflecting the higher accuracy of the collected data by the ellipsometer. Psi and delta values are consistent with the reference database information for Ni (Table 14).

**Table 14:** XRD results at 240 °C/200 cycles of Ni deposition

MSE	6.104
Thickness 2: nickel (nm)	0.14 ± 0.018
Thickness 1: zinc oxide (nm)	34.29 ± 0.045
Average growth rate (Å/cycle)	0.007

At 260 °C, Fig. 6 shows that the thickness of ZnO decreased to 15.14 nm, whereas the thickness of Ni film increased to 0.21 nm. The MSE value is 4.75, revealing the increased accuracy of the acquired data. Psi and delta values remain consistent with the reference database values for Ni (Table 15). Table 15 shows the thickest Ni deposition, corroborating the EDS data in Table 5.

**Table 15:** XRD results at 260 °C/200 cycles of Ni deposition

MSE	4.750
Thickness 2: nickel (nm)	0.21 ± 0.010
Thickness 1: zinc oxide (nm)	15.14 ± 0.739
Average growth rate (Å/cycle)	0.0105

Based on Fig. 7, at 280 °C, ZnO thickness increased to 29.66 nm from the last trial, whereas Ni thickness decreased to 0.13 nm. Table 16 shows the MSE, thickness of Ni and ZnO films (nm), and average growth rate (Å/cycle). The MSE value is above 10, calculated at 12.289, indicating decreased accuracy. This finding is possibly due to the size of the fractionated sample within the ellipsometer, thereby reducing the surface area to be detected by the beam, which has a substantially larger diameter in comparison. Despite the slightly increased MSE value, the psi and delta values are within the reference range for Ni, according to its database.

**Table 16:** XRD results at 280 °C/200 cycles

MSE	12.289
Thickness 2: nickel (nm)	0.13 ± 0.067
Thickness 1: zinc oxide (nm)	29.66 ± 0.142
Average growth rate (Å/cycle)	0.0065

At 300 °C, Fig. 8 shows a reduction in ZnO and Ni film thickness to 0.04 and 9.54 nm, respectively. Table 17 reveals the MSE, the thickness of Ni and ZnO films (nm), and the average growth rate (Å/cycle). The MSE value is 1.362, revealing higher accuracy over previous trials. The psi and delta values are again within the reference Ni range, according to the database.

**Table 17:** XRD results at 300 °C/200 cycles

MSE	1.362
Thickness 2: nickel (nm)	0.04 ± 0.000
Thickness 1: zinc oxide (nm)	9.54 ± 0.016
Average growth rate (Å/cycle)	0.002

**Fig. 9:**
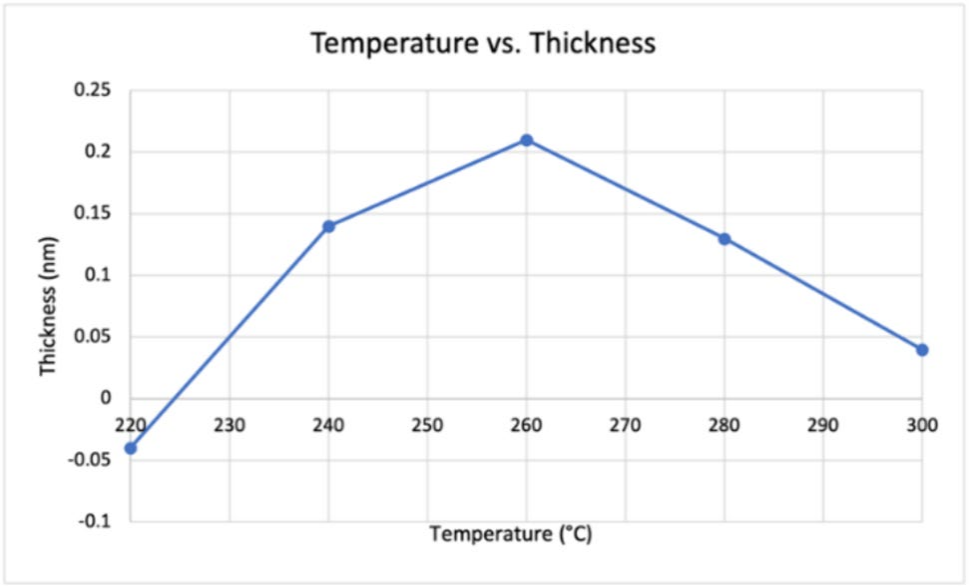
Graph revealing the temperature of Ni deposition versus Ni film thickness

The temperature and thickness of the Ni film show a directly proportional relationship; as temperature increases, thickness also increases. However, at 260 °C, the thickness of the Ni film starts to decrease, as shown in Fig. 9.

As previously mentioned, this finding may be due to the placement of the substrate within the ALD chamber because some regions contain higher precursor exposure than others. Additionally, the laser beam within the ellipsometer may have a greater diameter than the width of the microscopic globular growth, thus making it incapable of identifying the Ni layers and calculating the film thickness.

### XPS Analysis

The binding energy vs. intensity was calculated using Kratos AXIS Supra XPS via monochromate Al K⍺ radiation. In XPS, X-rays (photons) are shot into a sample; when electrons in the sample absorb sufficient energy, they are ejected from the sample with a certain kinetic energy [16]. The energy of said electrons is analyzed by a detector, and a plot of these energies and the relative numbers of electrons is produced. Electrons of different energies follow different paths through the detector, enabling the detector to differentiate the electrons and produce the spectra [16]. Binding energy is the energy of an electron attracted to a nucleus; photon energy is the energy of X-ray photons used by the spectrometer; and kinetic energy is the energy used to eject electrons from the sample. XPS reveals a low probability that electrons under the surface of the sample will escape and become detectable. XPS also contains surface sensitivity, which is explained by the Beer–Lambert Law for inelastic electron scattering shown below [16]:

5 $${{I}}_{{{z}}} = {{I}}_{{{0}}} {\text{exp}}\left( {\frac{{ - {{z}}}}{{{{\lambda}} {\text{sin}}{{\theta}} }}} \right)$$

6 $${{\lambda}} = {\frac{{ - {{z}}}}{{{{\text{ sin}}}{{\theta} }{\text{ln}}\left( {\frac{{{{I}}_{{{z}}} }}{{{{I}}_{{{0}}} }}} \right)}}} $$

where *z* is the depth of atoms that are ejecting electrons, *I*_*z*_ is the intensity of electron emission from depth *z*, *I*_0_ is the intensity of electrons from surface atoms, *θ* is the trajectory angle of electrons with respect to the surface plane, and *λ* is the average distance between inelastic collisions of an electron. The intensity of the signal decays exponentially due to the increased depth below the surface. The escape depth is calculated as $\frac{1}{\text{e}}$, which is 36.8% of its original depth.

**Fig. 10:**
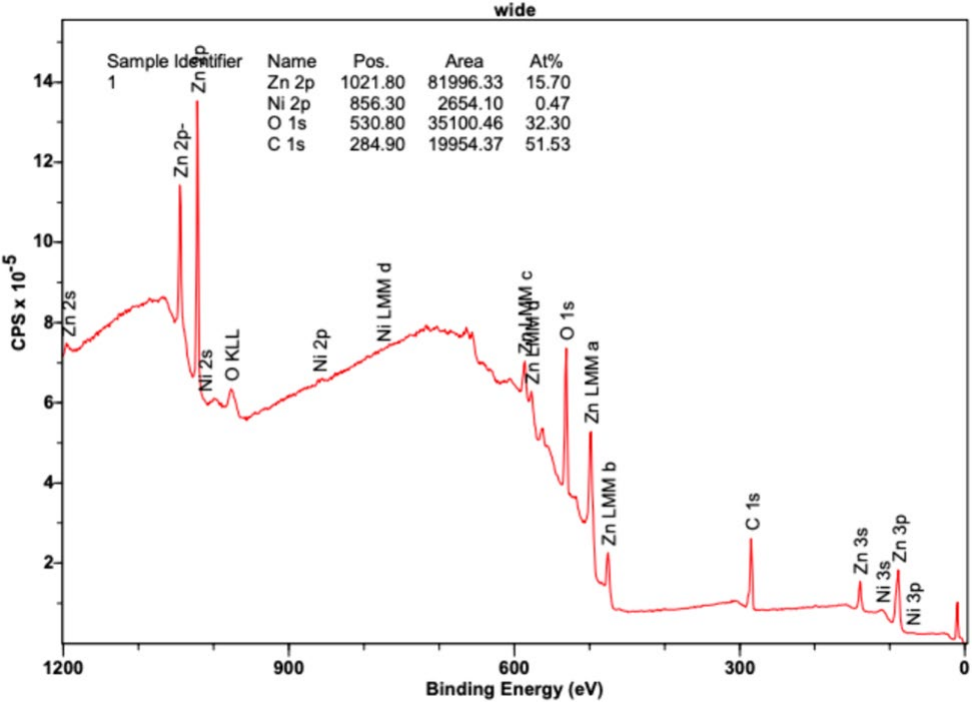
XPS results at 220 °C revealing binding energy (eV) versus intensity (counts per second—CPS)

Figure 10 displays binding energies from electrons in different orbitals. Their intensities reveal the atomic composition of the sample based on the amounts of each electron from different existing orbitals. Zn 2p orbitals reveal high intensities at position (eV) 1021.80, exhibiting a peak area of 81,996.33 counts per second (CPSeV) in its region and comprising an atomic concentration percentage of 15.70%. Zn MM orbitals a, b, c, and d, as well as Zn 3s, 3p, and 3d orbitals, reveal slight peaks between the 400–600 eV range, in accordance with the Zn XPS reference data. The O 1s orbital peak is also notable at 530.8 eV, revealing a large presence of oxygen in the sample with an area of 35,100.46 CPSeV and an atomic concentration percentage of 32.30%. Additionally, a heightened peak is observed at 284.9 eV, representing carbon (C 1s) with an area of 19,954.37 CPSeV and an atomic concentration percentage of 51.53%. Based on the larger sized peaks for Zn and oxygen, a notable presence of ZnO is found throughout the sample, revealing uniform deposition. Small amounts of Ni were detected as slight peaks in the 2p orbital and revealed at 856.3 eV, comprising an area of only 2654.10 CPSeV and an atomic concentration of 0.47%. Remarkable small, almost negligible peaks were also detected in the Ni 3s and 3p orbitals. Based on the results, Ni did not reveal a notable presence on the sample and did not have a conformal deposition.

**Fig. 11:**
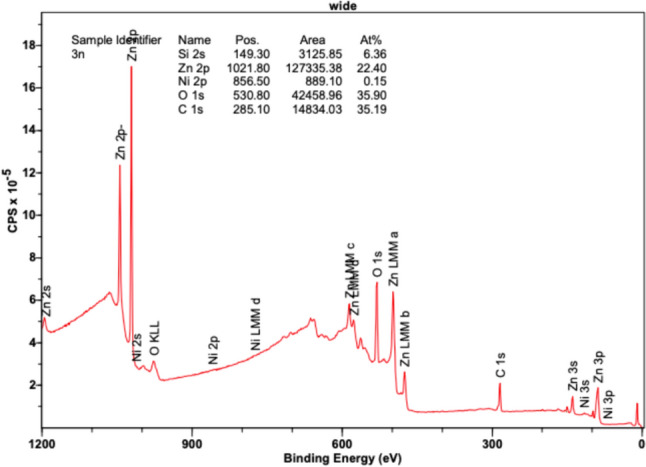
XPS results at 240 °C revealing binding energy (eV) versus intensity (counts per second—CPS)

Figure 11 shows Zn 2p orbitals with high intensities at position (eV) 1021.10, presenting an area of 101,807.88 CPSeV in its region. Zn MM orbitals a, b, c, and d, as well as Zn 3s, 3p, and 3d orbitals, reveal slight peaks between the 400–600 eV range, in accordance with the Zn XPS reference data. The O  1s orbital peak is also observed at 529.47 eV, revealing a large presence of oxygen in the sample with an area of 116,262.27 CPSeV. Another oxygen is also present in this sample; O 1s (possibly from OH) is detected at 530.98 eV with an area of 108,531.97 CPSeV. Additionally, a heightened peak is found at 285.82 eV, representing carbon (C 1s) with an area of 21,019.52 CPSeV. Based on the larger size of the peaks for Zn and oxygen, a notable presence of ZnO is observed throughout the sample, revealing conformal deposition. Small amounts of Ni, although higher than in the previous experiment, were detected as slight peaks in the 2p orbital and were revealed at 855.0 eV, comprising an area of only 5234.06 CPSeV. Remarkably small, almost negligible peaks were also detected in the Ni 3s and 3p orbitals. The results showed no notable Ni presence or conformal deposition on the sample.

**Fig. 12:**
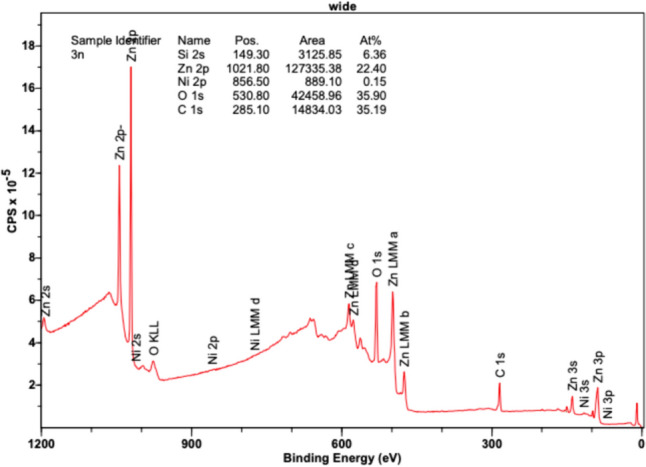
XPS results at 260 °C revealing binding energy (eV) versus intensity (counts per second—CPS)

Figure 12 reveals high intensities for Zn 2p orbitals at position (eV) 1021.80, exhibiting an area of 127,335.38 in its region and comprising an atomic concentration percentage of 22.40%. Zn MM orbitals a, b, c, and d, as well as Zn 3s, 3p, and 3d orbitals, reveal slight peaks between the 400–600 eV range, in accordance with the Zn XPS reference data. The O 1s orbital peak is also notable at 530.8 eV, revealing a large presence of oxygen in the sample with an area of 42,458.96 CPSeV and an atomic concentration percentage of 35.90%. Additionally, a heightened peak is observed at 285.10 eV, representing carbon (C 1s) with an area of 14,834.03 CPSeV and an atomic concentration percentage of 35.19%. Based on the larger size of the peaks for Zn and oxygen, a notable presence of ZnO was observed throughout the sample, revealing uniform deposition. Limited amounts of Ni were again detected as slight peaks in the 2p orbital and were revealed at 856.5 eV, comprising an area of only 889.10 CPSeV and an atomic concentration of 0.15%. Minute peaks were also detected in the Ni 3s and 3p orbitals. Based on the results, Ni did not reveal a substantial presence in the sample and did not have homogeneous deposition. A small presence was recorded; however, at 260 °C, the second highest abundance of Ni was observed using XPS.

**Fig. 13:**
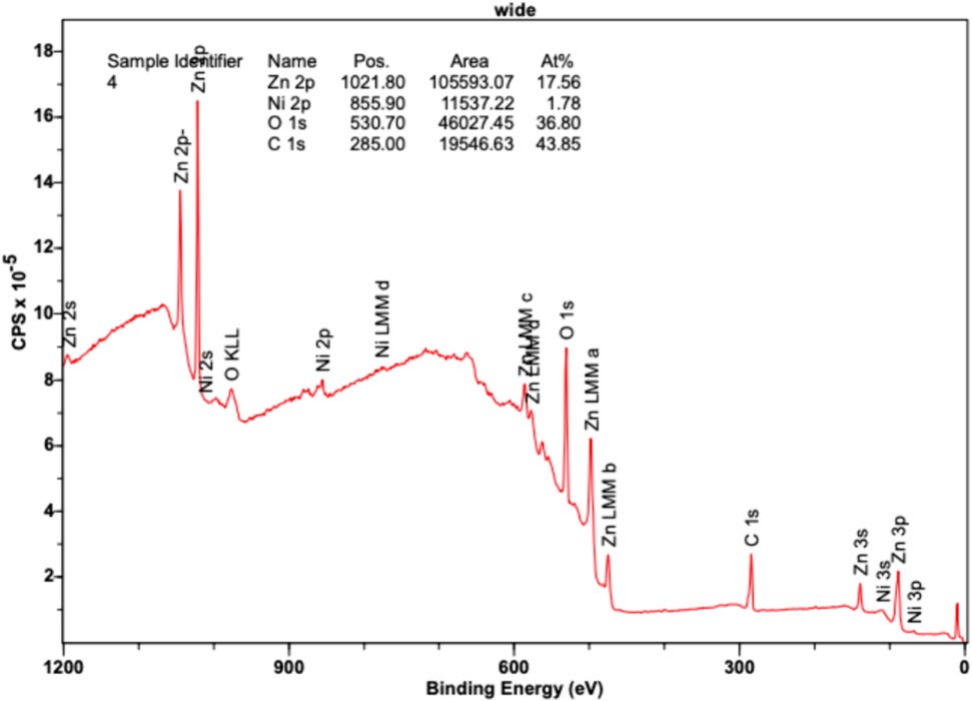
XPS results at 280 °C revealing binding energy (eV) versus intensity (counts per second—CPS)

Figure 13 also shows high intensities for Zn 2p orbitals at position (eV) 1021.80, exposing an area of 105,593.07 CPSeV in its region and comprising an atomic concentration percentage of 17.56%. Zn MM orbitals a, b, c, and d, as well as Zn 3s, 3p, and 3d orbitals, reveal slight peaks between the 400–600 eV range, in accordance with the Zn XPS reference data. The O 1s orbital peak is also notable at 530.70 eV, revealing a large presence of oxygen in the sample with an area of 46,027.45 CPSeV and an atomic concentration percentage of 36.80%. Additionally, a heightened peak was found at 285.00 eV, representing carbon (C 1s) with an area of 19,546.63 CPSeV and an atomic concentration percentage of 43.85%. Based on the larger size of the peaks for Zn and oxygen, a notable presence of ZnO was observed throughout the sample, revealing uniform deposition. Small amounts of Ni, although higher than the previous three experiments, were detected as slight peaks in the 2p orbital and were revealed at 855.90 eV, comprising an area of only 11,537.22 CPSeV and an atomic concentration of 1.78%. Insignificant peaks were detected in the Ni 3s and 3p orbitals. Based on the results, Ni was not prevalent in the sample and did not maintain consistent growth throughout the Si wafer.

**Fig. 14:**
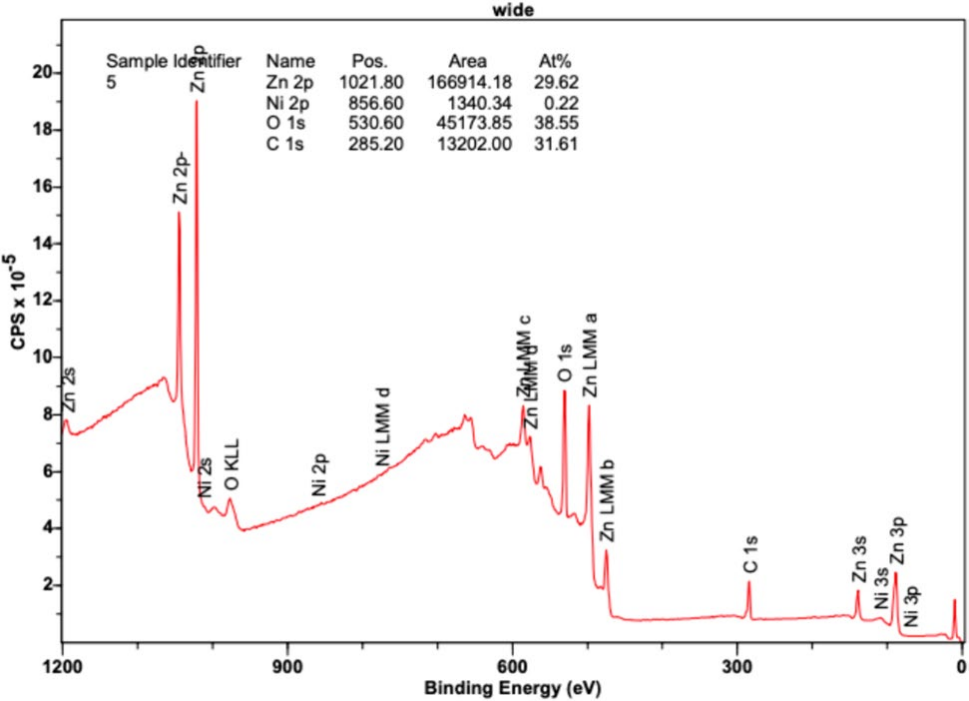
XPS results at 300 °C revealing binding energy (eV) versus intensity (counts per second—CPS)

Figure 14 displays Zn 2p orbitals with high intensities at position (eV) 1021.80, presenting a peak area of 166,914.18 CPSeV in its region and comprising an atomic concentration percentage of 29.62%. Zn MM orbitals a, b, c, and d, as well as Zn 3s, 3p, and 3d orbitals, reveal slight peaks in the 400–600 eV range, in accordance with the Zn XPS reference data. The O 1s orbital peak is also notable at 530.60 eV, revealing a large presence of oxygen in the sample with an area of 451,737.85 CPSeV and an atomic concentration percentage of 38.55%. Additionally, a heightened peak was observed at 285.20 eV, representing carbon (C 1s) with an area of 13,202.00 CPSeV and an atomic concentration percentage of 31.61%. Based on the larger size of the peaks for Zn and oxygen, a notable presence of ZnO was observed throughout the sample, revealing uniform deposition. Small amounts of Ni, which demonstrate a reduction from previous trials, were detected as slight peaks in the 2p orbital and were revealed at 856.60 eV, comprising an area of only 1340.34 CPSeV and an atomic concentration of 0.22%. Additionally, diminutive peaks were detected in the Ni 3s and 3p orbitals. Based on the results, Ni maintained a minor appearance on the sample, thereby lacking conformal deposition. A small presence was recorded; however, 300 °C revealed the highest abundance of Ni using XPS.

## Conclusions

Based on the data, 260 °C revealed the highest deposition rate across many regions of the silicon wafer atop ZnO. Characterization methods, including XRD, ellipsometry, and SEM, present supporting data for an ideal temperature of 260 °C. XPS revealed slightly higher deposition at 300 °C, with an increased intensity of 856.6 eV of Ni (displayed in Fig. 14) when compared to an intensity of 856.5 eV at 260 °C (displayed in Fig. 12). As previously mentioned, this finding may be due to the placement of the substrate within the ALD chamber because some regions contain higher precursor exposure than others. In the aforementioned analysis, the laser beam within the ellipsometer possibly obtains a greater diameter than the globular growths, which renders a difficult Ni thin film thickness reading. Across the wafer, varying weight concentrations were recorded, ranging from 0.43% to 42.82% (obtained from EDS on SEM). Notably, the optimal dose time of reactant A (Ni(dmamb)_2_) is 1000 ms, while that of reactant B (hydrogen gas) is 6 ms. The purge times for stages A and B are optimum at 10,000 and 120,000 ms, respectively. The cycle count was also increased to 200 from the previous 100 to help raise deposition via nucleation.

Ni deposition previously displayed an insignificant growth rate at < 1% composition. This finding may be due to minimal pulse heights of approximately 5 mTorr above the inactive gas chamber at 630 mTorr because the Ni(dmamb)_2_ is a low-vapor pressure precursor. Ni deposition revealed a higher growth rate at increased temperatures; however, some trials remained < 1%. Small regions of higher temperature trials revealed > 1% composition using EDS. According to SEM high-resolution images, no conformal deposition was detected across the silicon wafer. However, ZnO revealed slightly more uniformity throughout the regions, albeit imperfect. Diminutive globular growths were prevalent across the substrate. The results displayed < 1% composition of Ni; thus, thickness is also < 1 nm according to the ellipsometer data. Moreover, these results remained consistent with XRD and XPS data, revealing the presence of minimal Ni atop the ZnO adhesion layer at temperatures of 220–300 °C.

Overall, increased temperatures appear to maximize Ni growth because this condition may be a more suitable environment. Ni is an electropositive metal that tends to donate electrons and form positively charged cations. Remarkably, few reagents are capable of transforming Ni ions into pure Ni metal because the chemically reducing agent must donate electrons to the metal ions themselves. This process is usually performed at extremely high temperatures due to sufficient kinetic energy, breaking the strong bonds between lattices of valence electrons that metal ions typically form within their structure. Utilizing H_2_ gas is another method used to mitigate the issue due to the unreactive surfaces of metals. Upon reacting a metal oxide with an organometallic compound, such as ZnO with Ni(dmamb)_2_, a subsequent reaction, in which hydrogen radicals are used for the reduction of Ni metal to create thin films on a surface, occurs.

## Data Availability

The authors declare that all data supporting the findings of this study are available within the article.
